# Supplementary material for: A Clinical and Economic Comparison of Cell-Based Versus Recombinant Influenza Vaccines in Adults 18–64 Years in the United States
Source: Vaccines (Basel). 2024 Oct 26;12(11):1217. doi: 10.3390/vaccines12111217 (PMC11598601; doi:10.3390/vaccines12111217)
Supplement: Supplementary file 1 [file vaccines-12-01217-s001.zip › vaccines-3232885-supplementary.pdf]

Supplementary Information

A.

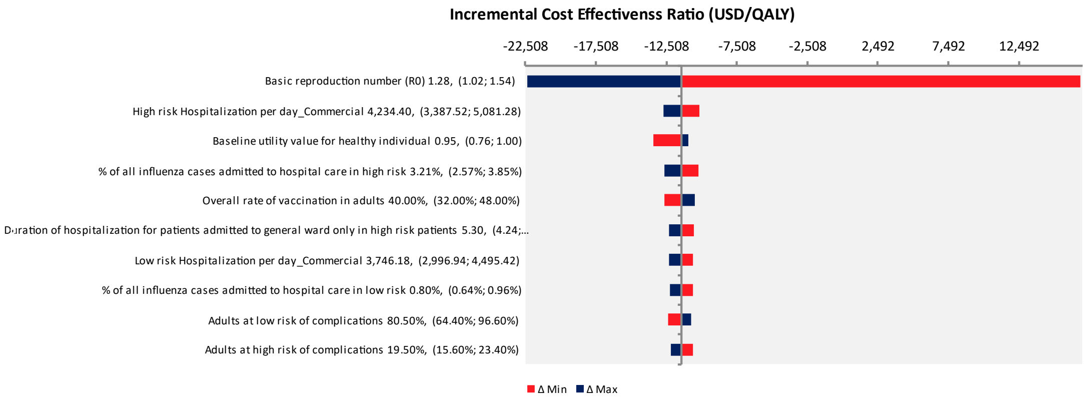

B.

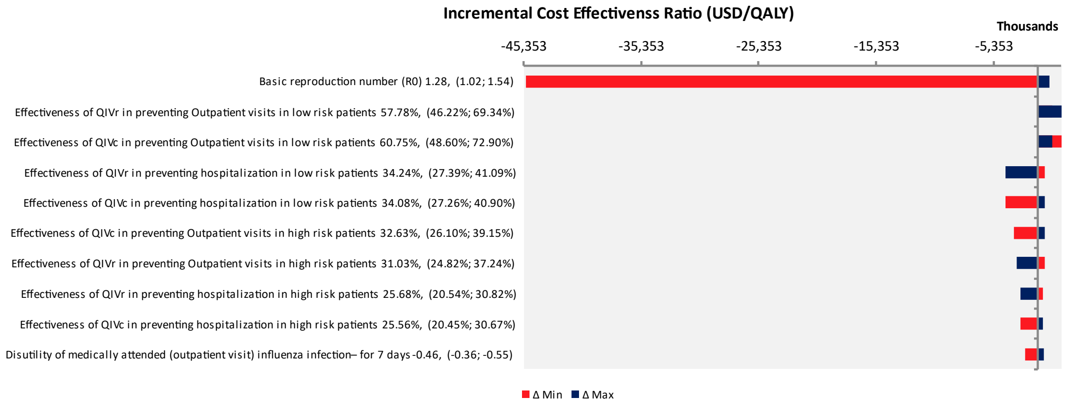

Figure S1: One-way sensitivity analysis tornado diagram for base case analysis (season 2018-2019) for cell-based vaccine versus (A) no vaccine and (B) recombinant vaccine from the US commercial perspective.

Table S1: Contact matrix between age groups (Mossong 2008)[20]

	**Age 0-19**	**Age 20-64**	**Age 65+**
**Source case: Age 0-19**	41.2%	53.4%	5.3%
**Source case: Age 20-64**	14.7%	78.4%	6.9%
**Source case: Age 65+**	12.1%	62.7%	25.2%

Table S2: Influenza-related medical encounter visits/admissions averted by cell-based and recombinant vaccines compared to no vaccination (season 2018-2019).

	**Cell-based vaccine**	**Recombinant vaccine**	**Difference cell-based vs. recombinant**
Outpatient clinic visits averted	-26,993,917	-26,558,981	-434,936
ED visit averted	-1,758,548	-1,761,064	2,516
Hospitalizations averted	-707,214	-707,920	705

Table S3: Cost-effectiveness results for scenario analyses from US commercial payer perspective (season 2018-2019)

	**Cell-based vs. no vaccine**	**Recombinant vs. no vaccine**	**Cell-based vs. recombinant vaccine**
**Scenario 1: 10-year time horizon**			
Cost of vaccines and administration	$5.026 B	$7.850 B	-$2.824 B
OP visits costs	-$2.891 B	-$2.844 B	-$46.582 M
ED visits costs	-$3.288 B	-$3.293 B	$4.705 M

Total hospitalizations costs	-$13.083 B	-$13.096 B	$12.695 M
Long term sequelae	-$0.964 B	-$0.965 B	$0.870 M
Total Cost	-$15.201 B	-$12.349 B	-$2.852 B
Total QALYs	1,899,410	1,897,620	1,789
ICER ($/QALY)	Dominant	Dominant	Dominant
**Scenario 2: Vaccine effectiveness estimates from 2019-2020 season**			
Cost of vaccines and administration	$5.026 B	$7.850 B	-$2.824 B
OP visits costs	-$2.870 B	-$2.861 B	-$8.469 M
ED visits costs	-$3.291 B	-$3.312 B	$20.704 M
Total hospitalizations costs	-$13.154 B	-$13.149 B	-$5.078 M
Total Cost (US$)	-$14.289 B	-$11.473 B	-$2.817 B
Total QALYs	1,244,323	1,244,009	314
ICER ($/QALY)	Dominant	Dominant	Dominant
**Scenario 3a: Cost-effectiveness results in adults at low risk of developing complications**			
Cost of vaccines and administration	4.046 B	6.319 B	-2.273 B
OP visits costs	-2.330 B	-2.290 B	-$40.344 M
ED visits costs	-2.307 B	-2.311 B	$3.701 M
Total hospitalizations costs	-5.263 B	-5.269 B	$5.813 M
Total Cost (US$)	-5.854 B	-3.550 B	-$2.304 B
Total QALYs	935,469	933,880	1,589
ICER ($/QALY)	Dominant	Dominant	Dominant

Scenario 3b: Cost-effectiveness results in adults at high risk of developing complications			
Cost of vaccines and administration	$980.078 M	$1.531 B	-$550.694 M
OP visits costs	-560.843 M	-554.605 M	-6.237 M
ED visits costs	-981.685 M	-982.689 M	1.005 M
Total hospitalizations costs	-7,820.215 M	-7,827.098 M	6.882 M
Total Cost (US$)	-8.383 B	-7.834 B	-549.044 M
Total QALYs	309,414	309,188	226
ICER ($/QALY)	Dominant	Dominant	Dominant

Abbreviations: B, billion; ED emergency department; ICER; incremental cost-effectiveness ratio; M, million; OP, outpatient; QALY, quality-adjusted life year.
